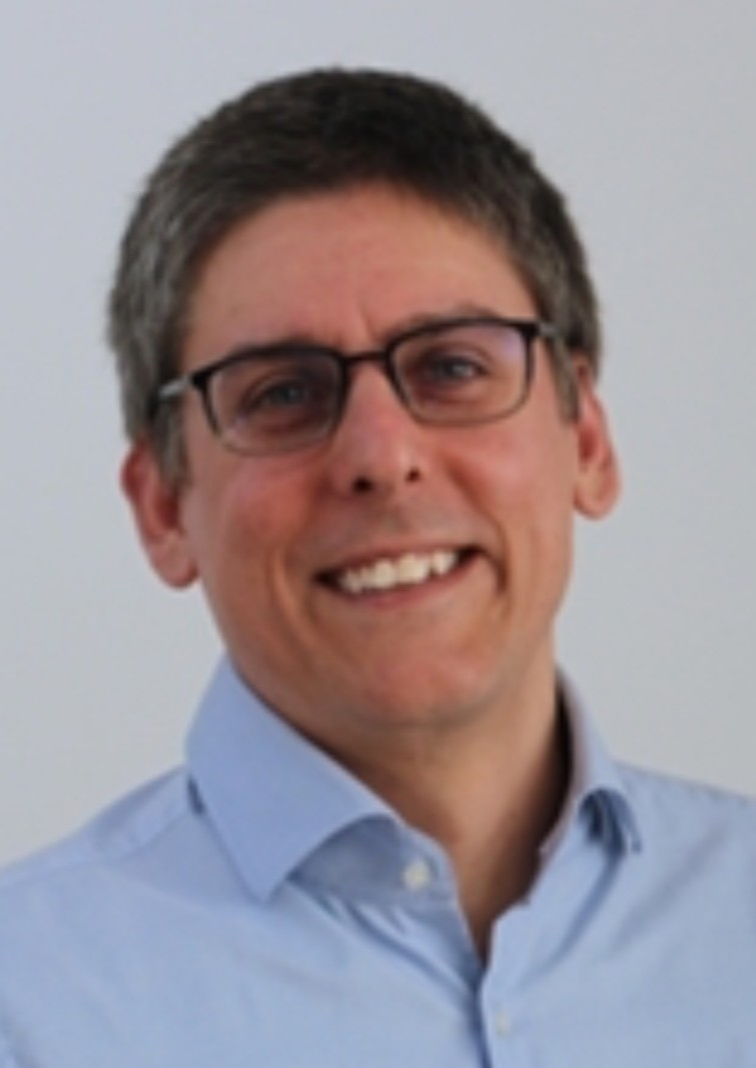# Spectro‐Microscopic Techniques for Studying Nanoplastics in the Environment and in Organisms

**DOI:** 10.1002/anie.202210494

**Published:** 2022-11-24

**Authors:** Laurens D. B. Mandemaker, Florian Meirer

**Affiliations:** ^1^ Inorganic Chemistry and Catalysis Debye Institute for Nanomaterial Science Universiteitsweg 99 3584 CG Utrecht The Netherlands

**Keywords:** Detection, Micro-Spectroscopy, Microscopy, Nanoplastics, Spectroscopy

## Abstract

Nanoplastics (NPs), small (<1 μm) polymer particles formed from bulk plastics, are a potential threat to human health and the environment. Orders of magnitude smaller than microplastics (MPs), they might behave differently due to their larger surface area and small size, which allows them to diffuse through organic barriers. However, detecting NPs in the environment and organic matrices has proven to be difficult, as their chemical nature is similar to these matrices. Furthermore, as their size is smaller than the (spatial) detection limit of common analytical tools, they are hard to find and quantify. We highlight different micro‐spectroscopic techniques utilized for NP detection and argue that an analysis procedure should involve both particle imaging and correlative or direct chemical characterization of the same particles or samples. Finally, we highlight methods that can do both simultaneously, but with the downside that large particle numbers and statistics cannot be obtained.

## Graphical abstract

Nanoplastics (NPs) pose a threat to the environment and human health but are challenging to detect and characterize in environmental samples. This Minireview covers established imaging and characterization techniques, with a focus on micro‐spectroscopic techniques. More exotic advanced techniques to measure NP morphology and chemical fingerprints simultaneously are discussed, and challenges in detection of NPs using high‐resolution imaging are outlined.

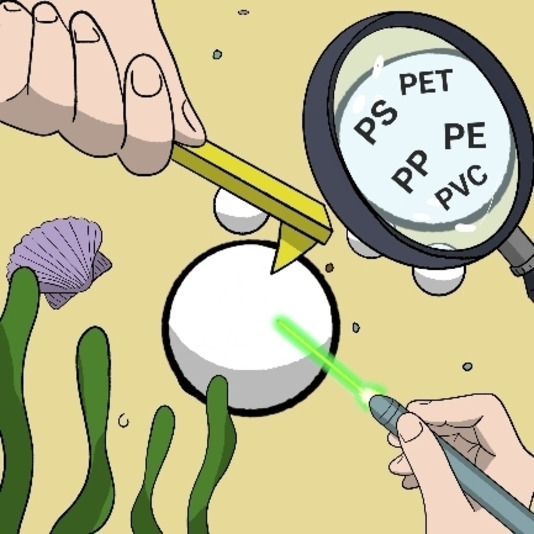

## 1 Introduction

Since the 1950s, our consumer market has been dominated by polymer‐based products and applications. Plastics, increasingly generated during and after the Second World War, have developed at a pace previously unseen for other man‐made products.[^1^, ^2^, ^3^] Their unpaired versatility, in terms of tunable physical properties, great resistivity against varying conditions including temperature, humidity and, for example, microwaves, varying flexibility and stiffness, as well as cheap production costs have led to the fact that plastic products are still the most optimal product choice for many different types of applications in daily life, if viewed from a product‐usage perspective.^[4]^ However, concerns are growing on how the plastic market affects our planet in hazardous ways, considering that 90 % of all plastics are single‐used and discarded, while only 9 % are successfully recycled.[^1^, ^5^] Large amounts of plastic waste are stored in landfills and end up in the oceans, either through rivers or directly. It is estimated that between 4.8 to 12.7 million tons of plastic end up in the ocean every year.^[6]^

In that context, additional concerns were raised in recent years as it was estimated that 99 % of the plastic waste that ends up in the ocean is missing; that is, not found back on the ocean surface.^[7]^ One hypothesis explaining this mismatch is the degradation of bulk plastic products into smaller pieces, namely micro‐ or nanoplastics. The definition of the size of these smallest plastic size fractions is still under debate; for this work, we consider microplastic (MP) particles as 5 mm–1 μm in diameter, and nanoplastic (NP) as smaller than 1 μm in diameter. The current general consensus is that plastics are weathering and/or degrading into smaller and smaller pieces, mainly by UV radiation, chemical, mechanical, and biodegradation.[^7^, ^8^, ^9^] In reality it is unquestionably a combination of all of these factors, depending on the location, plastic type, and conditions. Furthermore, these smaller plastics are not only found in the ocean but are also present in air, in soil, in food and, as recently reported by Leslie et al., even in our blood.[^10^, ^11^, ^12^, ^13^, ^14^] Assessing the potential health risks these particles might pose in a specific framework is a herculean task, as it needs to take into account an almost infinite amount of combinations of polymer types, sizes, shapes, and chemical surface groups.^[15]^ It is, nevertheless, an important task as MPs have been shown to have potentially negative health effects on fish, algae, mice, and human cells.[^12^, ^16^, ^17^, ^18^, ^19^, ^20^]

**Figure 1:**
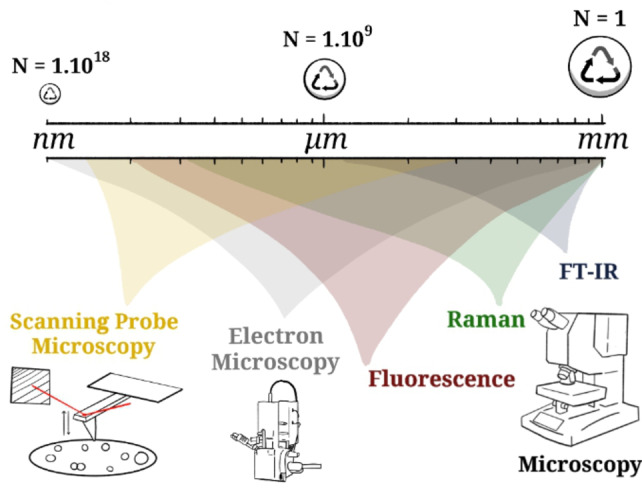
Representation (spheres are not to true scale) of the size range from microplastics (MPs) towards nanoplastics (NPs) and the number of particles given in *N*, which make up the same volume assuming constant density. Different micro(spectro)scopies will be discussed in this Minireview, which are displayed here with the relevant range of their detection limits.

Although the risks, presence, and characterization of MPs present in the environment are slowly being established, related to their size, NPs represent an additional level of difficulty for detection, characterization and, in turn, risk assessment. This leads to a characterization gap: NPs are so small that light‐based microscopes using ultraviolet, visible, or infrared light are unable to image them because they are smaller than the diffraction limit (Figure 1). Furthermore, their particle number‐to‐mass ratio increases significantly when the particles become smaller. To put this into numbers (Figure 1), a spherical particle with a diameter of 1 mm has the same mass as 1 billion particles with 1 μm diameter, or a trillion particles with a diameter of 100 nm; it is, to the best of our knowledge, not yet clear whether total mass or particle numbers are more significant in risk assessment concerning health effects of NPs. This leads to another challenge in NP characterization, because even a very large number of NPs might have such a small mass that it cannot be simply detected by mass characterization techniques (that is, (pyrolysis) GC‐MS, LC‐MS, TGA‐MS, and ICP‐MS) without proper preconcentration steps. However, simple preconcentration results in accumulation of contaminations if not done selectively, which highlights the necessity that extreme precautions must be taken to avoid contamination for mass‐based quantification techniques. Finally, the characterization gap is further increased by the chemical composition of NPs, as they mainly consist of carbon, hydrogen, and oxygen. In that sense, NPs are very similar to the organic matter that often accompanies them in environmental or tissue samples, which in turn limits the analytical methods based on element contrast that may be employed.

While the major health effects of NPs, if any, are yet unknown, they will arguably depend on concentration, particle size, (surface) chemistry, and chemical composition in terms of additives and polymer type. NPs behave differently from MPs due to their small weight and increased surface area. It was, for example, shown that they are transported differently through water bodies and aggregate differently than MPs because, for NPs, these processes are dominated by Brownian motion and structural layer force.^[21]^ Furthermore, they are expected to have a different effect on, for example, the intestinal tract, as the smaller NPs could cause more oxidative stress and inflammation. Similar effects are expected for the reproductive and nervous system, where NPs are considered to be of higher toxicity than MPs.^[22]^ Other work drew identical conclusions for human lung epithelial cells, which could eventually lead to cell death.^[23]^ Particle size might be a problem in general, as NPs can diffuse through membranes that are impenetrable to MPs, as was shown in the work of Mattsson et al., for example. In that study, zooplankton with NPs were fed to fish, and it was shown that the NPs were transferred to the fish and able to cross the blood–brain barrier, leading to brain damage and behavioral disorder of the fish.^[24]^

Although the health effects of NPs are still poorly understood, an increasing number of studies are reporting on the detection of NPs from the environment, mostly centered around aquatic environments. However, NPs were found in drinking water,^[25]^ atmospheric samples,^[26]^ infant feeding bottles (although it was suggested that these were contaminations and additives, namely fatty acids),[^27^, ^28^] human amniotic fluid (although its origin is uncertain),^[29]^ and soil.[^30^, ^31^] Note that there is much more work reported on, for example, polar ice, river water, soil, Artic ice, and other environments, that relies heavily on mass‐based characterization. This technique has established itself as the most facilitated method: ≈80 % (18 out of 23 we found) of these reports only use mass‐based characterization techniques. While accurate background checks and analysis fingerprints were properly established (for most, some did not verify their technique using spiked samples), actual particles were not imaged (or only a few), and studies generally lack a (resulting) particle size distribution (PSD). The reason for this is that determining size distributions requires a method to count NPs, which is non‐trivial, especially for non‐mono disperse PSDs and samples that also contain non‐plastic particles in similar size regimes, which is typically the case for environmental samples. While (multistage) size fractionation using filters of various suitable pore sizes can help to separate size fractions, mass‐based methods are most powerful in detecting the presence and quantifying the amount of polymers in a (size fraction of a) sample, but can never conclude whether the determined mass is a result of a few larger particles or, in the other extreme, due to a high number of (decomposed) polymer chains.

Therefore, we argue that for a complete characterization of environmental NPs, both imaging methods and chemical characterization (at the single‐particle level) should be performed additionally. Potential contaminations in the form of larger plastic particles dominating the detected mass or, on the other hand, dissolved polymers, can be ruled out if additional characterization is done on the single‐particle level, and ideally complemented by mass‐based quantitative methods. Herein, we review the techniques that are applied and/or potentially applicable to measure NPs (<1 μm) to obtain both morphological and chemical information at the single‐particle level. Elegant review articles on the characterization of MNPs already exist, which put a large focus on MPs and mass‐based characterization techniques; these are outside the scope of this article, as we specifically focus on micro‐spectroscopic imaging of NPs.[^32^, ^33^, ^34^, ^35^] The advantages and disadvantages of the different techniques highlighted in Figure 1 will be discussed, with examples from reported work but also with practicalities, advantages, and disadvantages for characterizing NPs. Finally, we suggest additional characterization methods that have not (or have only scarcely) been applied to measure NPs but are promising tools to measure NPs directly in tissue or other complex matrices.

## 2 Nanoplastic Characterization Techniques

Microscopic measurements on NPs are often challenging because methods can either achieve a high spatial resolution, but obtain no chemical information, or they can obtain a chemical fingerprint, but at the cost of a larger spot size. We focus first on “single” microscopy techniques that excel in one of the two, and then assess correlative studies and techniques that can achieve both.

### 2.1 Electron Microscopy

Electron microscopy is a frequently used characterization tool with very high spatial resolution. Whereas scanning electron microscopy (SEM) yields high‐resolution images of the surface morphology of the sample, transmission electron microscopy (TEM) looks through the samples, creating a shadow (projection) image of the whole sample. Both methods are widely applied within the field of MNP imaging, as they allow for a relatively quick and easy assessment of particle size, shape, and quantity. For this purpose, SEM or TEM has been used to image and visualize (spiked) NPs in various settings; for example, in plants,[^36^, ^37^] from medical grade face masks,^[38]^ under simulated environmental weathering conditions,^[39]^ in human cells,^[40]^ from river water,^[41]^ in sand water extracts,^[42]^ in algae,^[43]^ and many more.

**Figure 2:**
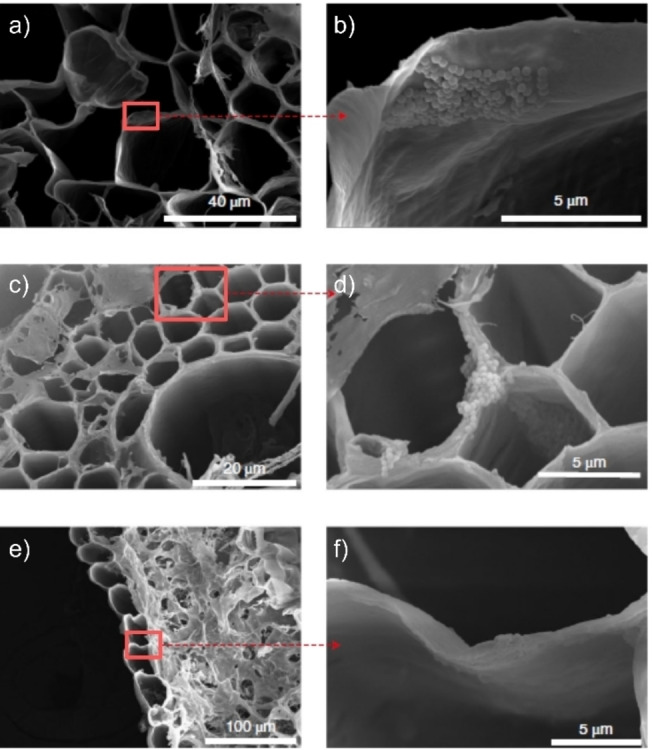
a)–d) Scanning transmission electron microscopy (STEM) images of wheat root and e, f) leaf after exposure to Eu‐doped polystyrene (PS) nanoplastics (NPs), 200 nm in size, wherein agglomerates of the particles are found predominantly in the roots but also in the leaves. The red boxes and arrows indicate the magnified regions, respectively. Adapted with permission from ref. [36]. Copyright 2022, Nature Publishing Group.

In a specific example, Luo et al. doped commercial polystyrene (PS) NPs (200 nm) with the europium chelate Eu‐β‐diketonate to create traceable NPs, which were provided to wheat and lettuce plants. The use of such labeled particles in spiking and exposure approaches is highly efficient, as it allows for tracing and imaging of the particles in the studied matrix without the need for digestion, preconcentration/filtration, and work‐up steps. (Fluorescently labeled particles will be discussed in detail below.) After proper incorporation, the authors used high‐angle annular dark‐field (HAADF) scanning transmission electron microscopy (STEM) to visualize and characterize the labeled particles. A common concern in labeling particles and exposure studies is the potential leaching of the marker into the matrix, leading to either false positives or false negatives. In the work of Luo et al., the particles were exposed to a medium simulating the conditions found in plant cells, and only a low amount of leaching was observed, which could not lead to Eu aggregation, and thus, false positives. Using a mm‐range field of view (FOV), the labeled particles were traced back mainly to the roots and found in limited amounts in the shoots, imaged with fluorescence microscopy. To confirm the presence of the spherical particles, SEM was used to image a root and leaf for both plants, as represented in Figure 2 for the wheat plant. Within the plant structure, agglomerates of Eu‐PS particles were found mostly on the xylem and cell walls of the cortex tissue of the wheat roots (Figure 2a–d), although some particles were spotted in the leaves as well (Figure 2e, f). This work elegantly demonstrates the power of combining microscopy on labeled particles using a larger FOV, focusing on the exposure and tracing of the particles, while high‐resolution microscopy is used to magnify and confirm the actual presence of the (here spherical) particles.

Electron microscopy techniques are essential in the characterization of NPs, as they are convenient, relatively fast, and accessible tools with high (compared to most other techniques in this review) spatial resolution. Furthermore, TEM can be used to measure particles in tissue or other solid matrices, although chemical discrimination will remain difficult. To overcome this, particles used for spiking or exposure studies can be stained, doped, or labeled in another way, to allow for elemental analysis (utilizing, for example, energy dispersive X‐ray (EDX) analysis), and to provide an optional extra confirmation in tracing the particles throughout samples via electron microscopy. However, for irregularly shaped, non‐doped NPs—as they are expected to occur in the environment—this approach will not allow detection and identification of such particles.

### 2.2 Fourier Transform Infrared Microscopy

Vibrational spectroscopy is a powerful tool to characterize polymers. In terms of chemical characterization, it might be the most accessible and revealing method to look at the type of polymer, potential degradation (for example, due to the formation of oxygen‐rich species), and organic impurities/contaminations. However, spectroscopy on samples containing NPs is often challenging as the most relevant measurements are performed either directly in the sample matrix or after retrieving the particles from the matrix. Then, (leftover non‐ or partially digested) organic material will become a large disturbing factor in the vibrational spectrum, as organic matter consists of similar chemical groups as the polymers, making it practically impossible to filter out the spectrum purely corresponding to the NP particles. There are reports using FT‐IR spectroscopy to measure the interaction between NPs and microalgae;[^44^, ^45^] however, based on this technique alone it is too difficult to identify mixtures of plastics and non‐plastics, and discriminate between types of plastics within such mixtures. However, if exclusively and well‐defined NPs are tested under degradative conditions (which do not “contaminate” the IR spectrum), FT‐IR spectroscopy still proves to be a powerful tool in characterizing the state of the NPs, as demonstrated by Liu et al., who studied PS NPs before and after UV exposure and reported the formation of carbonyl groups after UV irradiation.^[46]^

**Figure 3:**
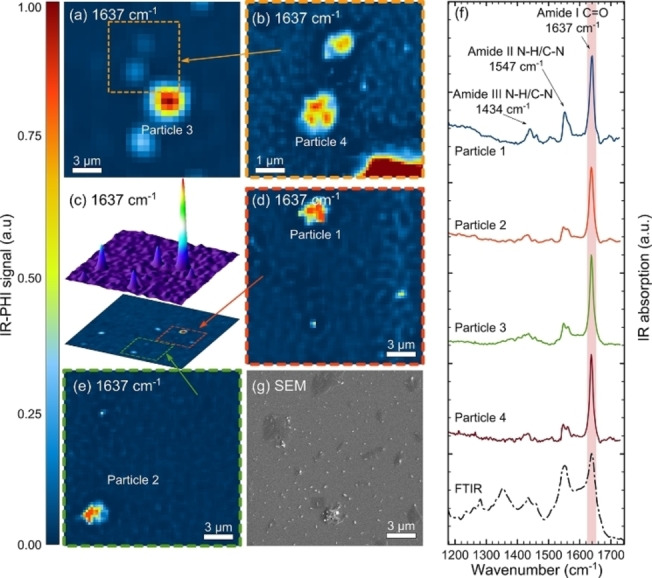
a)–e) Infrared photothermal heterodyne imaging (IR‐PHI) on steeped tea bags (at 95 °C), showing the intensity maps for the 1637 cm^−1^ C=O vibration. Note that the orange, green, and red boxes define the magnified scan areas. f) The specific spectra recorded on the labeled particles highlight the spectral components (representing Nylon) measured at such a high spatial resolution on particles ≤3 μm. g) A correlative scanning electron microscopy (SEM) micrograph confirms the MNPs presence and morphology. Reproduced with permission from ref. [50]. Copyright 2021 American Chemical Society.

A straightforward next step is FT‐IR microscopy; however, the spatial resolution is, in principle, limited by the wavelength of the IR light, which effectively results in achievable spatial resolutions around 10 μm for transmission‐ or reflection‐based measurements. Attenuated total reflection (ATR)‐IR spectroscopy uses the generated evanescent wave to measure IR “into” the sample, fixated on an ATR crystal, wherein both the depth into the sample and the spatial resolution (when an ATR module is combined with FT‐IR microscopy) are pushed towards 3 μm depending on the wavelength measured.^[47]^ Therefore, many publications can be found that use FT‐IR as a powerful tool to map and verify the chemical nature of MPs.^[48]^ This has not been done for NPs, although there are studies where MPs had been measured with FT‐IR microscopy and which claim that both NPs and MPs were detected; however, these reports will not be discussed here. Different from conventional IR microscopy, approaches to “super‐resolution” IR imaging and spectroscopy seem promising for the detection of NPs.^[49]^ Kniazev et al. demonstrated the potential power of the “super‐resolution” IR absorption technique called infrared photothermal heterodyne imaging (IR‐PHI).^[50]^ By irradiating the sample with infrared light, small temperature differences are detected using a second continuous wave (CW) probe laser. Using such an approach, the spatial resolution of the IR maps is improved towards ≈300 nm; that is, similar to the resolution of Raman imaging (vide infra), and thus enabling the measurement of nanosized polymers.[^51^, ^52^] The authors demonstrate the possibilities of this technique on PMMA microspheres, and then applied the method to detect i) Nylon MNPs generated from tea bags steeped at 95 °C (Figure 3) or steeped at 25 °C, and ii) rubber particles found in bulk road dust.

### 2.3 (Surface‐Enhanced) Raman Spectroscopy

Raman spectroscopy and microscopy is performed using lasers that utilize smaller wavelengths than IR; thus, the spatial resolution is increased (and dependent on the laser applied). A generic spatial resolution of ≈300 nm is achieved using a 532 nm laser but, as Raman is less sensitive than IR micro‐spectroscopy, it can be difficult to obtain a proper vibrational spectrum for small (individual) particles. Many reports have overcome this general limitation by using surface‐enhanced Raman spectroscopy (SERS), where metallic surfaces with rough or nanostructured morphology are used to create hotspots in which the Raman signal is amplified by a factor of up to 10^10^.^[53]^ Thus far, different surfaces have been reported and used to measure various NPs, as is summarized in Table 1.

**Table 1:** Overview of reports utilizing surface‐enhanced Raman spectroscopy (SERS) to detect (spiked) nanoplastics (NPs).

NP types^[a]^	Sample	SERS substrate	Enhancement factor	Ref.
PS	Spiked water	Ag nanoparticles on Si wafer	Uneven	[54]
	Spiked lake water	Ag nanoparticles with KI	2.30×10^4^ ^[b]^	[55]
	Spiked water and sea water	Ag nanoparticles with NaCl	4×10^4^ ^[b]^	[56]
	Spiked bottled, tap, and river water	Nanowell patterned Ag film on SiO_2_ substrate	2.3×10^8^ ^[c]^	[57]
	Spiked water	Ag nanowires on regenerated cellulose	1.8×10^7^	[58]
PS/PMMA	Spiked water	Klarite substrate	172^[b]^	[26]
PS/PET	Air	Klarite substrate		[26]
PS/PET	Spiked water	Au nanoparticles on glass	445.7^[b]^	[59]
PE	Bottles and cups under irradiation	CuO/Ag nanoparticles	–	[60]
PS^[d]^	Exposed bivalves	PS‐coated Au nanostars^[d]^	–	[61]

[a] Key: polystyrene (PS), polymethylmethacrylate (PMMA), polyethylene terephthalate (PET). [b] The highest reported, but for one specific particle size. Other particle sizes had lower enhancement factors. [c] Calculated on measurements performed on *p*‐ATP molecules instead of on the NPs themselves. [d] The plastic and metal particle are the same: PS is grown around a SERS nanostar.

The majority of the work presented in Table 1 focused on spiked samples of well‐defined NPs instead of detecting environmental NPs. This is no coincidence, as most of the authors mention in their discussion section that, although SERS is a very sensitive technique, sample dispersion on the substrate remains a challenge and the overall sensitivity, and thus detection limit established, is not yet sufficient to measure NP concentrations in environmental samples. Additionally, as elaborated before, the presence of additional organic features would make the resulting spectra very challenging to interpret.

### 2.4 Raman Microscopy

Raman imaging presents many additional benefits compared to Raman spectroscopy and IR micro‐spectroscopy—mostly due to its higher spatial resolution—making Raman microscopy one of the workhorses in the characterization of MPs. However, for NP mapping, the spatial resolution is still not sufficient for detecting the smaller NP size fractions and sensitivity is low (see section 2.3). There are reports in which Raman microscopy was used to measure 100 nm particles, but this required extensive image processing and still does not allow a mapping of the true morphology of the NP.^[62]^ Furthermore, the resulting spectrum of such a particle has a very low signal‐to‐noise ratio; only a single peak could be found within the spectrum, which means it cannot be used as a characteristic fingerprint for the plastic. Therefore, applying this to real‐life environmental samples is still challenging and will not provide (more detailed) information on particle morphology or chemical identification. However, if the particles are large enough, Raman imaging has this advantage, and particle morphology can be linked to spectral characteristics, thereby allowing it to selectively analyze the plastics and distinguish them from other material, even in complex matrices. An example can be found in the work of Gillibert et al., where the authors spiked distilled water and seawater with PS, PMMA, and PE NPs (and PS, PP, PMMA, PE, PVC, and PET MPs) and were able to identify them with Raman micro‐spectroscopy.^[63]^ The challenge of a low Raman scattering signal was overcome by using optical tweezers, which captured and trapped particles of the same size after field‐flow fractionation, allowing for size‐dependent micro‐spectroscopic analysis; this is enabled by using an optical trap, generated due to the momentum exchange between light and particles during the scattering interaction when using tightly focusing laser beams. For NPs, this is only possible with a high numerical aperture (NA) and laser power, as the Brownian motion of the particles can destabilize the optical trap. Applying this technique resulted in successful measurements of the spectra of individual 100 nm sized NPs.

**Figure 4:**
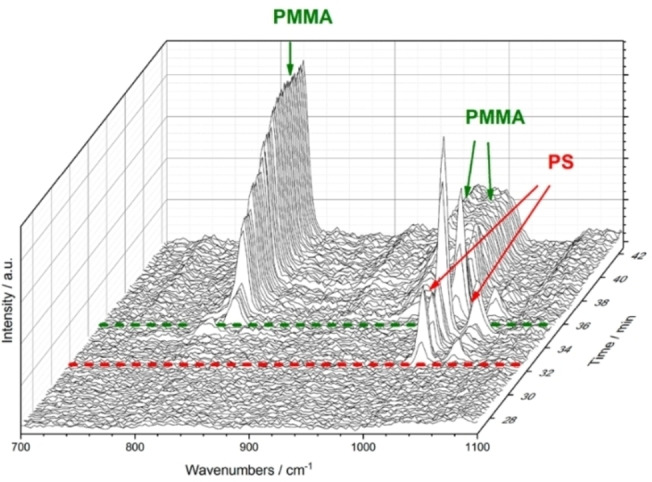
3D Raman spectroscopy plot over time, demonstrating the proper asymmetric flow field flow fractionation (AF4) and subsequent chemical identification, in which the smaller (350 nm) PS particles come off first, as seen from their characteristic Raman bands, followed by the larger (500 nm) PMMA particles. Adapted with permission from ref. [64]. Copyright 2020 American Chemical Society.

Schwaferts et al. presented another report in which optical tweezers were used that additionally increased the practicalities of using Raman micro‐spectroscopy for the characterization of plastic particles in mixtures. The authors performed online Raman micro‐spectroscopy coupled to a field flow fractionation setup (which also included UV and multiangle light scattering).^[64]^ A clear separation and proper identification was shown for a mixture of PS (350) and PMMA (500 nm) particles (Figure 4). Using this combined approach, particles could be detected in the size range from 200 nm to 5 μm and with a minimum concentration of approximately 1 mg L^−1^. This concentration limit is still high, but the elegant pre‐separation before applying Raman micro‐spectroscopy, combined with size characterization via light scattering, allows the assessment of specific NP‐rich aqueous environmental samples or of samples with more complex matrices after a careful pre‐concentration step.

### 2.5 Fluorescence Microscopy

Fluorescence microscopy is widely applied to trace the uptake of NPs in different organisms, cells, or other media. The spatial resolution of the method is relatively high, and it is possible to measure <100 nm fluorescently labeled NPs when combined with confocal scanning (confocal fluorescence microscopy (CFM)).

All this can be done in a non‐destructive manner, which is why this technique has been the most popular in the field of NP tracing using fluorescent particles.[^66^, ^67^] Fluorescent NPs are available commercially, although the market, and resultingly also the research using them, is heavily dominated by PS particles.

This lack of commercially available NPs other than PS particles led to a surge in research on the synthesis of fluorescent particles, and several groups reported protocols to generate traceable particles, either by integrating fluorescent organics or metals.[^24^, ^68^, ^69^, ^70^] Fluorescent particles have been used to track the capture of NPs in nanocellulose networks,^[71]^ test the stability of NPs in digestion protocols,^[72]^ and to study the uptake in organisms such as maize plants,^[73]^ marine larvae,^[68]^ diatom algae,^[69]^ acorn barnacle,^[70]^ daphnia magna,[^74^, ^75^, ^76^] mouse brain cells,^[77]^ zebrafish,[^78^, ^79^] and freshwater mussels.^[80]^ It should be noted that leaching of the fluorescent dye (even from commercial particles) and autofluorescence in organic matrices might make the interpretation of results challenging or misleading, as was shown when the fluorophores alone agglomerated within zebrafish larvae and the fish larvae itself displayed green autofluorescence.^[81]^ Therefore, we argue that measuring blanks and additional tests to quantify the amount of leaching from the fluorescent particles in the matrices studied are a must. Another possibility we see is to further improve the synthesis procedures to yield fluorescent NPs with covalently bound fluorophores, or to make use of the autofluorescence of the plastics (if they have any), as demonstrated by Lionetto et al. in a recent report.^[82]^ However, one must also take into consideration the effect of the fluorophore on the plastic properties. If the dye is added as an additive, it might act as, for example, a plasticizer, whereas binding it covalently might change the intrinsic order of the polymer. Although fluorescence microscopy is one of the most suitable techniques to identify NPs in spiked samples directly with relative ease, the obvious but main limitation is that this can only be done for particles (generated and) added to the samples, and not for environmental or real‐life NPs.

### 2.6 Correlative Approaches

As mentioned in the earlier sections, often a single technique lacks the ability to obtain all the specifics that are needed to assess both morphology and chemical nature of (individual) NPs. However, when combined, the available characterization toolbox can measure more NP properties with specifically optimized parameters for the individual techniques. A first correlative approach would be to directly combine two techniques, which allows for a direct combination of imaging (with high spatial resolution) and chemical characterization. This is a well‐established approach for MPs, in which SEM and Raman micro‐spectroscopy are combined,^[83]^ and it was also used for spiked seawater and amniotic fluid samples.^[29]^ Another way to correlate two different techniques is finding back the same FOV on a sample but recorded by different techniques. This can be done using sophisticated “nano‐GPS” systems, which support automatization of finding, for example, a specific particle.^[84]^ A more manual approach is to use patterned samples, such as a grid, or markers on the sample carrier, and then use these markers to find and image the same particles using different microscopies. The patterned sample carriers can, moreover, serve as wells that specifically “capture” NPs of relevant sizes, thereby facilitating faster characterization as NPs are located (or accumulated) in certain regions on the sample carrier. This was done by Valsesia et al., where SEM was used to quickly identify which wells were occupied by material, and these wells were then mapped by confocal Raman micro‐spectroscopy, measuring the PS/Si bands to judge if a well was occupied by a PS particle or not.^[85]^ With such approaches the development and production of suitable substrates for selective accumulation of NPs in certain regions of the substrate is essential, and more work in this area will certainly push the field forward for measuring real environmental samples after digestion.

### 2.7 Scanning Probe Microscopy

Scanning probe microscopy (SPM) techniques, and more specifically atomic force microscopy (AFM), uses a sharp tip on a cantilever to raster scan a surface and record the height difference due to the physical attractive and repulsive forces between the tip and the sample. The spatial resolution is, hence, not related to the diffraction limit, but to the tip radius and scanning environment. The high nm‐range resolution, which is rather easily obtained, comes at the price of a smaller field of view (FOV) that can be inspected with, for example, light‐based microscopy techniques. However, as the cantilever acts as a spring, recent years have shown the development of nanoindentation using AFM, providing information on physical characteristics such as stiffness and deformation, and the method appears to be the only tool capable of obtaining such important parameters for plastics at such small size scales.^[86]^ AFM has already been used to study NPs (uptake) in several reports.[^27^, ^82^, ^87^, ^88^]

An emerging technique based on AFM uses the same basic principle to measure sample morphology, but additionally performs IR spectroscopy by combining AFM with an IR source and using the AFM tip as an “antenna”. Essentially, the IR laser irradiates the surface under the AFM tip, leading to both a thermal expansion and a change in the dipole moment of the sample upon IR absorption. The IR source can be tuned through the desired spectral range, generating a wavelength‐dependent response in the sample. The induced physical effects are measured using either photothermally induced resonance spectroscopy, scattering scanning near‐field optical microscopy, or photoinduced force microscopy (PiFM). Excellent review articles are available explaining the working principle of these methods in‐depth.[^89^, ^90^]

**Figure 5:**
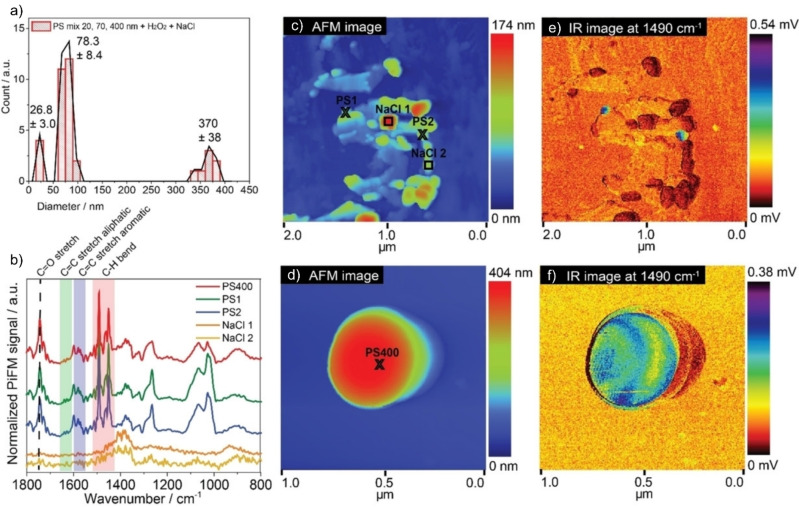
Photoinduced force microscopy (PiFM) results on a mixture of PS spheres in saline conditions, spin coated on a wafer. a) The different particle sizes are found using this approach, and even the smaller particles around 20 nm. b) PiF IR spectra are recorded on different features shown in the c), d) topology scans. e), f) The corresponding IR maps taken at 1490 cm^−1^, which represents the PS C=C stretch. The IR map (e) shows many features that give no IR intensity, also represented in the spectra in (b). These features are salt crystals, highlighting the strength of this tool to selectively trace and measure NPs. Furthermore the C=C stretching and aliphatic C=C stretching vibrations indicate degradation of the particles after exposure to these saline conditions. Reproduced from ref. [65].

As shown by ten Have et al., this so‐called AFM‐IR or nano‐IR technique (specifically, the authors used PiFM) facilitates the accurate detection of PS NPs down to 20 nm in size.^[65]^ The surface sensitivity highlighted a change of spectrum after H_2_O_2_ treatment of the (commercially obtained) PS beads. This is explained by sulfonate‐containing surfactants used to stabilize the particles: at first there were S=O stretching vibrations visible in the spectrum but, after the treatment, these bands had disappeared. Furthermore, the work demonstrated the power of the technique by measuring PS beads spin‐coated from saline solutions (Figure 5). First, the AFM was able to identify the different sizes of PS particles in the mixture. More interestingly, due to the IR signal also being recorded simultaneously, polymer particles could easily be identified even when attached within a larger agglomerate of NaCl (salt crystals). The spectrum revealed additional bands at 1750 cm^−1^, representing a C=O stretch, and at 1463 and 1640 cm^−1^, representing aliphatic CH_2_ bending and C=C stretching vibrations. These indicate that the PS spheres, after the removal of surfactant using H_2_O_2_, are prone to deactivate through oxidative and chain scission degradation.

Li et al. measured particles in retentates after elegantly filtering tap water over nanoporous filters with different pore sizes using a variety of techniques, including AFM‐IR.^[91]^ Although the particles measured with AFM‐IR seem to be more in the micro‐ than nanosize range, they used the spectral information to identify polyolefins, PA, PS, and PVC in tap water.

Merzel et al. showed that AFM‐IR could even be applied in tissue, although the probing depth is limited due to the high surface sensitivity of the technique.^[80]^ They dosed 1000 nm PS spheres into mussels and could easily identify the shapes with AFM, also obtaining the characteristic PS vibrations in the corresponding IR spectrum.

Bossers et al. studied nanosized PE domains while they were formed on the catalyst.^[92]^ Although not environmental NPs, the work presents a toolbox to study nanosized PE strings, including the PiFM method. The recorded IR spectra were analyzed using a multivariate curve resolution (MCR) approach, and allowed a determination of changes in the relative crystallinity of PE over time. The high spatial resolution combined with such spectral analysis is another example of how this technique could identify potential degradation mechanisms or material properties, such as crystallinity at the individual NP level.

Besides the IR‐based SPM techniques, Raman‐based techniques such as tip‐enhanced Raman spectroscopy (TERS) would be a promising technique to measure both particle morphology and chemical fingerprint. However, at the time of writing of this Minireview no work has been reported on the characterization of environmental NPs using TERS.

### 2.8 Other Micro‐Spectroscopic Techniques

**Figure 6:**
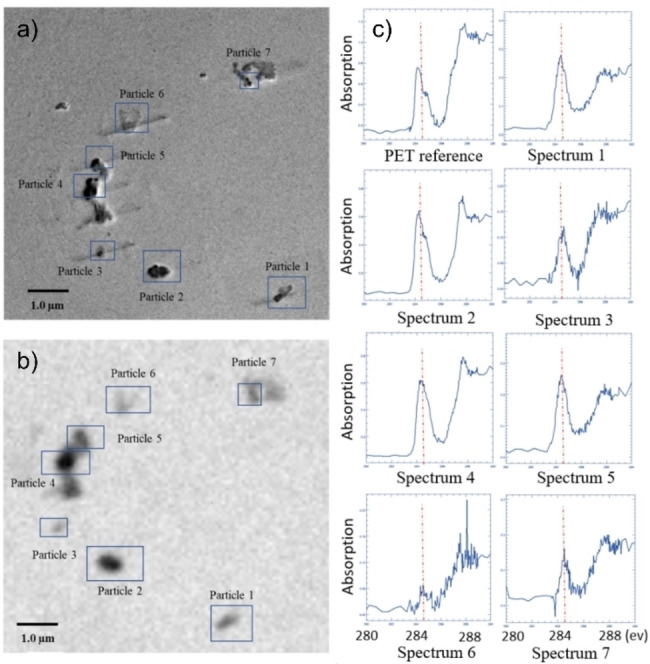
a) Transmission electron microscopy (TEM) and b) scanning transmission X‐ray microscopy (STXM) images of the same FOV on a washing sample grid. For all 7 particles c) the NEXAFS spectrum was measured and shown to correspond well with reference PET. Adapted from Ref. [93].

Besides the presented “conventional” methods to measure NPs, other techniques could be or have been shown to be capable of obtaining chemical information at a spatial resolution that makes them suitable or at least promising for measuring NPs. One example is scanning transmission x‐ray microscopy (STXM). Foetisch et al. present the technique applied to NP analysis, showing the spectra obtained when commercial NPs were measured using STXM.^[30]^ Furthermore, spiked samples and environmental samples (tea and soil) were measured, finding four different polymer types and different sizes. Yang et al. used STXM, amongst other techniques, to detect NPs released from washing textiles and found mostly PET NPs.^[93]^ Figure 6 displays the TEM image of a sample and the corresponding STXM image. In STXM the near‐edge X‐ray absorption fine structure (NEXAFS) of the sample can be measured by scanning the X‐ray energy of the incoming beam over the (here) carbon X‐ray absorption edge, providing information about chemical state and species of the plastic particle. Such spectra are presented in Figure 6c for the individual particles, showing good agreement with the PET reference sample. As STXM is a synchrotron‐radiation‐(SR) based method that requires access to a SR facility, particle counting at higher throughput will be very much restricted for this method. Nevertheless, it allows for the detection and characterization of single particles in terms of the size, shape, and chemical fingerprint, even in more complex sample matrices. However, the NEXAFS recorded for such samples will be harder to interpret due to contributions from any organic matter present in the sample, which is similarly the case for IR spectroscopy.

Another promising technique offers the strength of MS combined with imaging: time‐of‐flight secondary ion mass spectrometry (ToF‐SIMS). By using focused ion clusters, the samples are sputtered in a spatially resolved way and the generated ions are detected using a ToF detector measuring the ions with high *m*/*z* accuracy. This method is widely applied for polymers,^[94]^ and could become a more pivotal player in the NP research field as it can map individual mass fragments and gain detailed MS spectra; thus, it is also able to distinguish other (organic) matter on the sample surface.^[92]^ However, the spatial resolution limits its potential as a high imaging resolution yields pixel sizes of approximately 100 nm, but comes at the expense of spectral resolution and signal intensity. Using this (destructive) technique in a correlative way, as described earlier, would be greatly beneficial, by first imaging the particles with, for example, electron microscopy, and then recording their MS spectrum (and thus revealing more than just plastic identification) using ToF‐SIMS, as was demonstrated by Chou et al.^[95]^

Finally, combining spectroscopic identification and electron microscopy was also very recently demonstrated by Höppener et al. who utilized SEM with cathodoluminescence (that is, the light that is emitted when a material is excited by an electron beam) to show that HDPE, LDPE, PP, PA, PS, and PET all have characteristic luminescence spectra.^[96]^ This, combined with the high spatial resolution of SEM, should allow for the detection of different NPs, with expected challenges remaining in potential beam damage and a low sensitivity for smaller samples.

## 3 Conclusion

Although NPs potentially pose a serious threat to the environment and are subject to much ongoing research, a suitable, well‐defined toolbox of characterization techniques has not yet been established. Conventional techniques, such as IR or Raman microscopy, lack the spatial resolution to measure NPs of all size fractions, whereas electron microscopy or AFM provide sufficient spatial resolution but lack the ability for chemical identification, which is essential for the analysis of environmental samples. Therefore, correlative approaches seem most promising for advancing the field, together with more exotic micro‐spectroscopic techniques, such as AFM‐IR or STXM, which allow both the morphological and chemical characteristics of particles to be revealed at the nanoscale. Nevertheless, these imaging methods are often time‐demanding and simply unable to provide datasets containing high‐density information for a large number of particles or samples, which limits their power for providing statistically relevant particle‐size distributions, for example. Therefore, we argue that only a combination of such micro‐spectroscopic techniques, complemented by a mass‐based or particle‐number‐based technique (such as py‐GC‐MS or AF4‐MALS‐DLS) can provide a complete picture of both amount and identify of the NPs present. Therefore, current efforts focus on establishing protocols for sample pre‐processing, including filtration, digestion, and pre‐concentration, which can allow for such correlative analytical approaches. This work will face several challenges. For example, we expect that, especially in the smaller size range of NPs, mass‐based methods will already be at the lower limit of detection while the number of NPs is still too large to form a monolayer of well‐separated particles desired for efficient detection via AFM. The same holds true for samples where a large number of non‐plastic nanoparticles is present, which can cover the NPs on the sample carrier and interfere with both single‐particle identification via microscopy methods and the specific spectroscopic fingerprints used to characterize the NPs. Smart solutions for combining sample preparation and analytical methods are required, but we are confident that these will be developed in the coming years with the aim to increase the throughput of NP characterization and at some point even allow for their direct (that is, micro‐spectroscopic) detection within complex sample matrices.

## Conflict of interest

The authors declare no conflict of interest.

## Biographical Information

*Laurens D. B. Mandemaker received his B.Sc. and later M.Sc. degree in Chemistry from Utrecht University (The Netherlands). He did a Ph.D. under the supervision of Prof. Bert M. Weckhuysen in the Inorganic Chemistry and Catalysis Group at Utrecht University, focused on micro‐spectroscopic characterization of the growth, activity, and deactivation of metal‐organic framework films. He is currently a Postdoctoral Researcher focused on utilizing micro‐spectroscopy techniques as a toolbox to detect and characterize micro‐ and nanoplastics in various sample matrices*.

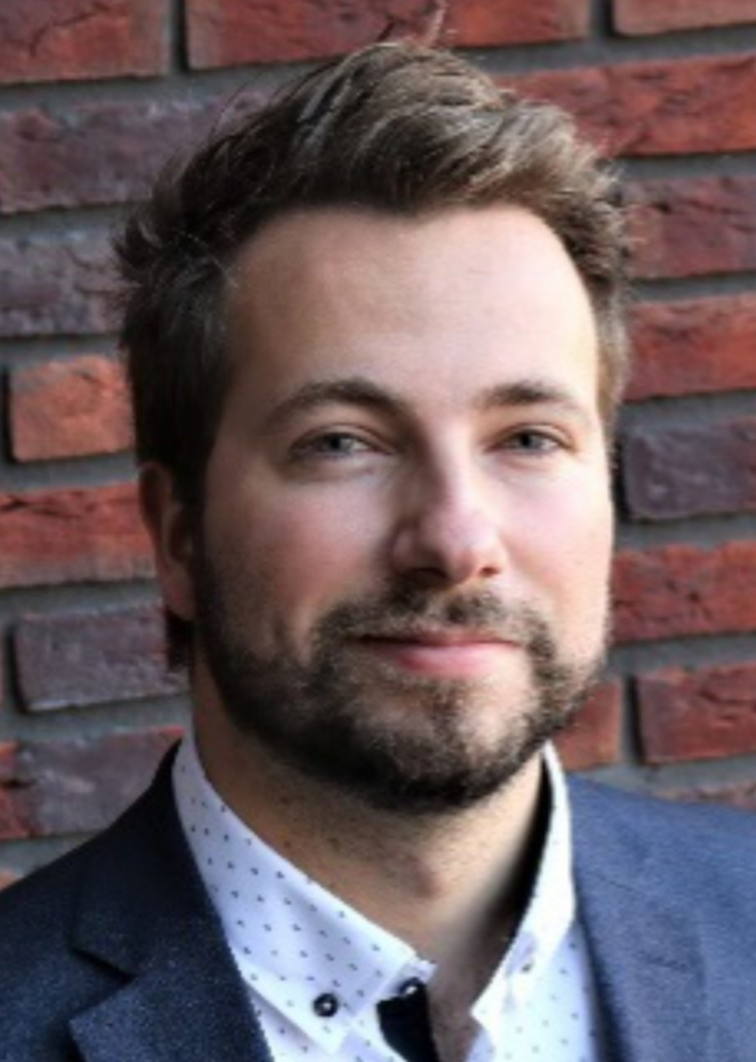

## Biographical Information

*Florian Meirer obtained his Sc.D. degree in technical physics from the TU Wien under supervision of Prof. Christina Streli. After postdoctoral stays at the Stanford Synchrotron Radiation Lightsource, USA, and the Fondazione Bruno Kessler, Italy, he moved to Utrecht to work on analytical methods for the characterization of solid catalysts and related nanomaterials. He is currently Associate Professor, and his research focuses on spectro(micro)scopy, data mining, and chemometrics in the fields of heterogeneous catalysis and environmental analysis*.